# Drug utilisation across occupations among pregnant women working in the healthcare and social assistance sector in France: a nationwide population-based study

**DOI:** 10.1136/oemed-2026-110920

**Published:** 2026-08-28

**Authors:** Mélanie Araujo, Justine Bénévent, Christine Damase-Michel, Yolande Esquirol, Anna-Belle Beau

**Affiliations:** 1 Hôpital Universitaire de Toulouse, CERPOP-SPHERE, Inserm UMR 1295, Université de Toulouse, Toulouse, France; 2 Hôpital Universitaire de Toulouse, Centre Régional des Pathologies Professionnelles et Environnementales, CERPOP-ARTERRE, Inserm UMR 1295, Université de Toulouse, Toulouse, France

**Keywords:** Health Personnel, Women, Epidemiology, Workers

## Abstract

**Objectives:**

Medication use during pregnancy is common, but patterns may differ across occupational subgroups, particularly in healthcare and social assistance where working conditions, education and health literacy vary. We aimed to compare medications dispensed to pregnant women across occupations within this sector.

**Methods:**

We conducted a nationwide population-based study using the French EDP-Santé database, linking occupational and socioeconomic data with healthcare reimbursement records. Pregnancies from 2009 to 2015 among women aged 15–55 years were identified. Medication dispensing during the year before and during pregnancy was described by occupational groups using the Anatomical Therapeutic Chemical classification. Comparisons were performed within healthcare occupations.

**Results:**

Among 321 569 pregnancies, 38 860 (12%) involved women in healthcare and social assistance. Before pregnancy, 91%–95% received at least one medication (mean: 8–12). Psychotropic medication (including antidepressants and benzodiazepines) dispensing was higher among hospital service workers (18% (95% CI 17% to 19%)), healthcare assistants (16% (95% CI 15% to 16%)) and social workers (14% (95% CI 13% to 15%)) than among physicians (11% (95% CI 10% to 13%)), rehabilitation professionals (10% (95% CI 8% to 12%)) and nurses (10% (95% CI 9% to 10%)). Similar patterns were observed for analgesic and musculoskeletal medications. During pregnancy, differences were less pronounced. Folic acid, recommended before and early in pregnancy, was lower among hospital service workers (30% (95% CI 29% to 32%)) than other healthcare workers (39% to 53%).

**Conclusion:**

Medication dispensing is common among pregnant healthcare workers, with marked variation across occupations. These differences may reflect disparities in working conditions or socioeconomic status, underlining the need for tailored maternal health support in specific occupational groups.

**WHAT IS ALREADY KNOWN ON THIS TOPIC**

- Women working in the healthcare and social assistance sectors are often exposed to challenging working conditions that may impact maternal health.
- In France, over 90% of pregnant women receive at least one medication during pregnancy (routine prenatal supplements, for acute conditions or chronic conditions).

**WHAT THIS STUDY ADDS**

- This is the first large-scale study to examine medication use across specific occupational subgroups in the healthcare and social assistance field.
- Occupations involving higher physical workload, psychosocial demands and shift work (such as hospital service workers and healthcare assistants) had significantly higher rates of psychotropic and musculoskeletal medication dispensing.

**HOW THIS STUDY MIGHT AFFECT RESEARCH, PRACTICE OR POLICY**

- There is a need for targeted preventive strategies for healthcare and social assistance workers, particularly in high-risk occupations like hospital service workers and healthcare assistants.

## Introduction

 Working conditions can affect maternal health during pregnancy through physically demanding tasks, irregular working hours and psychosocial stressors.^1–3^ The healthcare and social assistance sector is a particularly relevant context for study, as it has a high proportion of women, who account for over 70% of the workforce.^4^ This sector is also characterised by a wide range of occupations with heterogeneous working conditions (such as shift work, lifting weight, non-sitting working postures, working with radiation and infectious agents), as well as heterogeneous educational backgrounds and health-related knowledge. In addition, women working in this sector are in close contact with the healthcare system, which may influence their use of healthcare services.

The challenging working conditions faced by healthcare professionals, including night work, long working hours, lifting and carrying loads, and inappropriate postures have been associated with adverse pregnancy outcomes.^5–9^ However, most studies have focused on specific occupational groups, such as nurses or physicians, leaving other occupations underexplored. Moreover, little is known about the prenatal care and medication use among pregnant healthcare professionals.

Beyond pregnancy outcomes, working conditions may also influence healthcare utilisation and health-related behaviours. Evidence from other settings suggests higher psychoactive substance use among healthcare professionals. For instance, a US study reported signs of alcohol dependence in 15% of physicians versus 9% in the general population.^10^ A 2021 French survey highlighted inadequate care for depression among healthcare professionals, resulting in higher use of anxiolytics and hypnotics.^11^

However, broader data on prescription and dispensing of medications (including psychotropics, analgesics and non-steroidal anti-inflammatory drugs (NSAIDs)) during pregnancy are lacking. Moreover, recent and alarming reports on the working conditions of healthcare workers in France highlight the need to better understand the health of pregnant healthcare professionals.^12^

In this context, medication dispensing serves as an indicator not only of underlying health status but also for healthcare utilisation. Conceptually, one’s occupation is characterised by working conditions and socioeconomic status, which in turn affect maternal health and healthcare utilisation, and that is ultimately reflected in medication dispensing patterns. Given the descriptive nature of this study, analyses were primarily exploratory and consisted of comparisons of medication dispensing between occupational groups. The interpretation of the observed differences was guided by this conceptual framework, particularly considering the potential role of working conditions, as well as socioeconomic status.

This study aimed at evaluating medication dispensing before and during pregnancy among women working in the healthcare and social assistance sector, with a focus on occupational differences. We compared medication dispensing within this sector across different occupational groups, as well as between health sector and other sectors.

## Material and methods

### Data source

We used data from the EDP-Santé 2017, a French administrative database linking the Permanent Demographic Sample (EDP) from the French National Institute for Statistics and Economic Studies with the National Health Data System (SNDS).^13^ The EDP-Santé constitutes a representative sample of the French population, covering approximately 4% of the French population. Briefly, the EDP covers civil status, personal life (family situation), professional life (professional status, activity) and economic data (income, tax status), mainly derived from tax declaration and census data. The SNDS systematically records every healthcare encounter that generates a reimbursement under the universal health insurance scheme: outpatient care (medication dispensed, medical visits and procedures) and inpatient care (diagnoses and dates of hospitalisation). Over-the-counter (OTC) medications not reimbursed are not captured. Additional details are provided in the online supplemental methods 1.

### Pregnancy identification

Pregnancies occurring between January 2009 and December 2015 were identified using hospital discharge diagnoses and medical procedures recorded in the SNDS. The start of pregnancy was defined as the first day of the last menstrual period (LMP), estimated primarily from gestational age recorded during hospital stays, mainly based on the first-trimester ultrasound. When gestational age was missing, the median gestational age by pregnancy outcome observed in the general population was assigned, as done previously.^14^ Full algorithms and codes are detailed in the online supplemental table 1.

### Study population

We included women aged 15–55 years at the start of pregnancy. The study population was divided into two groups: (1) Healthcare and social assistance professionals; and (2) Non-healthcare professionals.

### Occupational classification

Occupation status during the periconceptional period (defined as the year of the LMP date) was mainly derived from employer declarations recorded in the EDP ‘active worker panel’, supplemented by census data. Occupations were identified using the level-four French occupational classification codes. We grouped into eight occupational groups the healthcare and social assistance professionals: (1) Physicians: medical physicians, dental surgeons and midwives; (2) Psychologists; (3) Nurses; (4) Social workers; (5) Rehabilitation professionals; (6) Healthcare assistants: nursing, dental or medical or psychologist assistants, and childcare auxiliaries; (7) Hospital service workers; and (8) Other healthcare professionals, not classified elsewhere. The socioeconomic status was defined using three occupational categories: executives, intermediate occupations and employees. Healthcare and social assistance occupations were grouped into these three categories as follows: executives (physicians and psychologists), intermediate occupations (nurses and social and rehabilitation professionals) and employees (healthcare assistants and hospital service workers). To construct comparison groups, all non-healthcare occupations falling within the same occupational categories were selected. Farmers, artisans and traders, company managers and labourers were excluded as they did not correspond to these categories. We also retrieved data on sector of employment (healthcare, social assistance and public administration) and employer type (public and private). Classification details are provided in online supplemental table 2.

### Medication exposure

Medication dispensing data were obtained from the SNDS and classified using the Anatomical, Therapeutic and Chemical (ATC) system. We examined all medications dispensed during the year preceding and throughout pregnancy. Medications were first described at ATC level 1, followed by analyses of selected classes of interest, including folic acid, psychotropic medications (benzodiazepines and antidepressants), analgesics, musculoskeletal medications and NSAIDs. ATC codes are listed in online supplemental table 3.

### Statistical analysis

The unit of analysis was the pregnancy episode. Continuous variables were described using means and SD, and categorical variables using percentages with 95% CI. Given the exploratory nature of the study, analyses consisted of stratified comparisons of medication dispensing percentages across occupational groups and within each occupational category, without multivariable modelling. Differences were considered statistically significant when there was no overlap between the 95% CIs. This conservative approach was chosen due to the multiple comparison groups and the challenge to define a single relevant reference group. Analyses complied with French data protection regulations, and results based on fewer than 10 individuals were not reported.

Data management and creation of study variables were performed using the framework Apache Spark in RStudio V.4.3.3 environment of the SparklyR package, facilitating the manipulation of massive data. Formal analyses were performed using SAS Enterprise Guide V.8.3 (SAS Institute, Cary, North Carolina, USA) and RStudio V.4.3.3. All analyses were carried out in a secure environment provided by the CASD (Centre d’Accès Sécurisé aux Données (Ref. 10.34724/CASD)).

## Results

### Characteristics of the study population

Among the 3732 088 individuals in the EDP-Santé, we identified 321 569 pregnancy episodes from 214 423 women aged 15–55 years with pregnancy onset between January 2009 and December 2015 (figure 1). Of these, 38 860 pregnancies (12%) occurred among women employed in the healthcare and social assistance sector, and 164 384 pregnancies among women employed in other sectors with comparable socioeconomic status.

**Figure 1:**
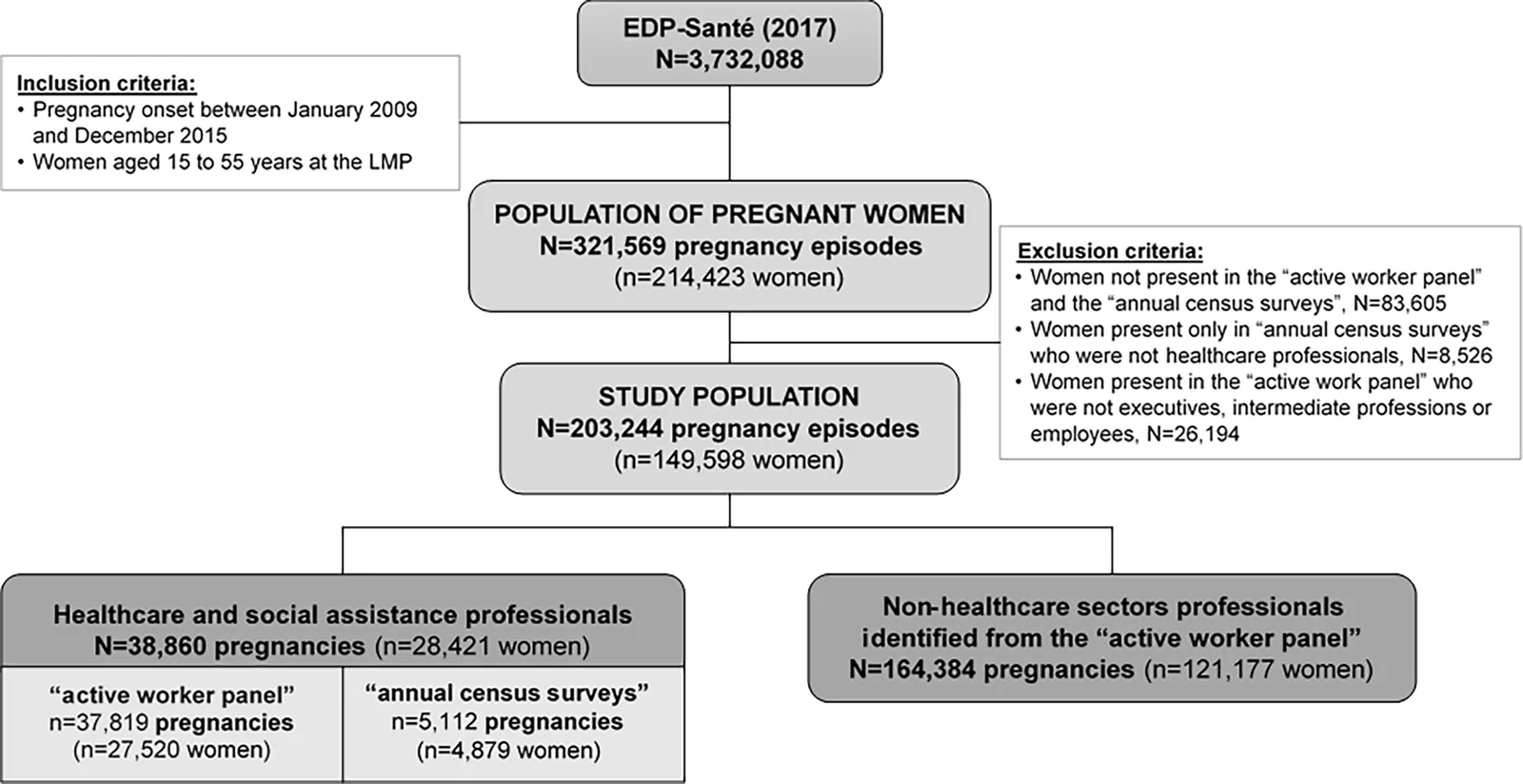
Flow chart describing the study population selection. A pregnancy can be identified in both the active worker panel and the annual census surveys, but was counted once in the data set. LMP, last menstrual period.

Within the healthcare and social assistance sector, 8% were executives, 48% intermediate occupations and 44% employees, compared with 15%, 20% and 65%, respectively, in other sectors. The healthcare and social assistance population included 11 004 (28%) healthcare assistants, 9521 (25%) nurses, 5602 (14%) hospital service workers, 4640 (12%) social workers, 2212 (6%) physicians, 959 (3%) rehabilitation professionals, 817 (2%) psychologists and 4105 (11%) other healthcare professionals such as pharmacists, medical technicians and opticians. Mean maternal age at pregnancy onset was 30.0±5.0 years, ranging from 28.2±6.1 years for hospital service workers to 32.3±4.0 for psychologists (table 1). Employment in the public sector varied across occupations from 35% among social work professionals to 81% among physicians.

**Table 1:** Population characteristics

	Physicians, n=**2212**	Psychologists, n=817	Nurses, n=9521	Social workers, n=4640	Rehabilitation professionals, n=959	Healthcare assistants, n=11 004	Hospital service workers, n=5602	Others, n=4105
Age at pregnancy onset (years)								
Mean±SD	31.3±4.3	32.3±4.0	30.2±4.5	31.0±4.5	30.6±4.1	29.7±5.2	28.2±6.1	30.0±4.5
<25	70 (3.2%)	5 (0.6%)	749 (7.9%)	238 (5.1%)	37 (3.9%)	1742 (15.8%)	1800 (32.1%)	369 (9.0%)
25–35	1655 (74.8%)	586 (71.7%)	7153 (75.1%)	3422 (73.8%)	756 (78.8%)	7216 (66.6%)	2862 (51.1%)	3050 (74.3%)
≥35	487 (22.0%)	226 (27.7%)	1619 (17.0%)	980 (21.1%)	166 (17.3%)	2046 (18.6%)	940 (16.8%)	686 (16.7%)
Sector of employment* (n (%))								
Health	1914 (89.7%)	349 (44.6%)	7819 (83.7%)	323 (7.2%)	452 (55.7%)	5221 (48.2%)	2347 (43.2%)	1416 (35.8%)
Social care	47 (2.2%)	299 (38.2%)	936 (10.0%)	2774 (61.6%)	277 (34.2%)	4355 (40.2%)	2917 (53.7%)	40 (1.0%)
Public administration	73 (3.4%)	94 (12.0%)	220 (2.4%)	925 (20.5%)	17 (2.1%)	575 (5.3%)	35 (0.6%)	37 (0.9%)
Others†	100 (4.7%)	40 (5.1%)	368 (4.0%)	480 (10.7%)	65 (8.0%)	675 (6.2%)	134 (2.5%)	2466 (62.2%)
Missing	*78*	*35*	*178*	*138*	*148*	*178*	*169*	*146*
Employer type (n (%))								
Public	1735 (81.3%)	389 (49.7%)	6130 (65.5%)	1556 (34.6%)	324 (40.0%)	5086 (46.9%)	2599 (47.8%)	633 (16.0%)
Private	399 (18.7%)	393 (50.3%)	3222 (34.5%)	2947 (65.4%)	487 (60.0%)	5748 (53.1%)	2841 (52.2%)	3330 (84.0%)
Missing	*78*	*35*	*169*	*137*	*148*	*170*	*162*	*142*

* Occupational sectors were categorised using the French A38 classification (derived from NACE Rev.2, European classification of economic activities).

† ‘others’ includes, for instance: wholesale and retail trade, administrative and support service activities, other service activities, education or legal, accounting and management activities.

### Medication dispensing before pregnancy

Overall 91%–95% of women were dispensed at least one medication in the year preceding pregnancy. The mean number of different medications ranged from 8 to 9 among physicians, nurses and rehabilitation professionals to 12 among hospital service workers (table 2). Physicians, nurses and rehabilitation professionals were dispensed significantly fewer medications than non-healthcare workers with a similar socioeconomic status, whereas hospital service workers were dispensed significantly more (table 2 and online supplemental table 4). Marked differences were observed across therapeutic classes (table 3 and online supplemental table 4). Nervous system medications (ATC N) were dispensed more frequently to hospital service workers (77% (95% CI 76% to 78%)), healthcare assistants (75% (95% CI 74% to 76%)), social workers (74% (95% CI 73% to 76%)) and psychologists (68% (95% CI 65% to 71%)) than to rehabilitation professionals (62% (95% CI 59% to 65%)), nurses (61% (95% CI 60% to 62%)) and physicians (60% (95% CI 58% to 62%)). The latter group was dispensed significantly fewer nervous system medications than non-healthcare workers with a similar socioeconomic status, while the former group was dispensed significantly more. For musculoskeletal system medications (ATC M), dispensing was significantly more frequent among hospital service workers (60% (95% CI 59% to 61%)) than among physicians (34% (95% CI 32% to 36%)). A similar pattern was seen for respiratory system medications (ATC R), with higher dispensing among social workers (54% (95% CI 53% to 56%)) compared with physicians (36% (95% CI 34% to 38%)). Conversely, blood and blood-forming organs medications (ATC B) were significantly more frequently dispensed to physicians (41% (95% CI 39% to 43%)), rehabilitation professionals (40% (95% CI 37% to 43%)) and psychologists (39% (95% CI 36% to 42%)) than to healthcare assistants (33% (95% CI 32% to 34%)) or hospital service workers (27% (95% CI 26% to 28%)). Physicians, rehabilitation professionals and healthcare assistants were significantly more likely to be dispensed ATC B medications than those with a similar socioeconomic status who did not work in the healthcare sector (table 3 and online supplemental table 4).

**Table 2:** Medications dispensed in the year prior to or during pregnancy by occupational groups of the healthcare sector

	Executives		Intermediate occupations			Employees		Others, n=4105
	Physicians, n=2212	Psychologists, n=817	Nurses, n=9521	Social workers, n=4640	Rehabilitation professionals, n=959	Healthcare assistants, n=11 004	Hospital service workers, n=5602	
Medications dispensed in the year prior to pregnancy								
At least one medication*	2017 (91.2%) (89.9% to 92.3%)	776 (95.0%) (93.3% to 96.3%)	8879 (93.3%) (92.7% to 93.7%)	4423 (95.3%) (94.7% to 95.9%)	891 (92.9%) (91.1% to 94.4%)	10 471 (95.1%) (94.7% to 95.5%)	5239 (93.5 %) (92.8% to 94.1%)	3855 (93.9%) (93.1% to 94.6%)
Number of different medications†	8.2±6.7 (7.9–8.5)	9.6±6.7 (9.1–10.1)	8.7±6.7 (8.5–8.7)	10.8±7.7 (10.6–11.0)	9.0±7.1 (8.5–9.4)	11.3±8.3 (11.0–11.4)	12.2±9.2 (11.9–12.3)	10.4±8.3 (10.1–10.7)
Medications dispensed during pregnancy								
At least one medication*	2076 (93.8%) (92.7% to 94.8%)	792 (96.9%) (95.5% to 97.9%)	8982 (94.3%) (93.9% to 94.8%)	4430 (95.5%) (94.8% to 96.0%)	919 (95.8%) (94.4% to 96.9%)	10 340 (94.0%) (93.6% to 94.4%)	5124 (91.5%) (90.7% to 92.2%)	3870 (94.3%) (93.5% to 95.0%)
Number of different medications†	7.2±5.1 (7.0–7.4)	8.3±5.5 (7.9–8.7)	7.2±4.9 (7.1–7.3)	8.3±5.5 (8.1–8.5)	7.4±4.8 (7.1–7.7)	8.2±5.7 (8.1–8.3)	8.1±6.2 (7.9–8.2)	8.2±5.8 (8.0–8.4)

* Expressed in n (%) and 95% CI.

† Expressed in mean±SD and 95% CI; differences between healthcare and assistance occupations were considered statistically significant when there was no overlap between the 95% CIs.

**Table 3:** Prevalence of medication dispensed by the first level of ATC classification in the year prior to pregnancy and during pregnancy by occupational groups of the healthcare sector

ATC classification	Executives		Intermediate occupations			Employees		Others, n=4105
	Physicians, n=2212	Psychologists, n=817	Social workers, n=4640	Rehabilitation professionals, n=959	Nurses, n=9521	Healthcare assistants, n=11 004	Hospital service workers, n=5602	
A: Alimentary tract and metabolism								
Dispensed in the year prior to pregnancy	1113 (50.3%) (48.2% to 52.4%)	477 (58.4%) (55.0% to 61.7%)	2762 (59.5%) (58.1% to 60.9)	495 (51.6%) (48.5% to 54.8%)	4699 (49.4%) (48.4% to 50.4%)	6565 (59.7%) (58.7% to 60.6%)	3446 (61.5%) (60.2% to 62.8%)	2353 (57.3%) (55.8% to 58.8%)
Dispensed during pregnancy	1746 (78.9%) (77.2% to 80.6%)	680 (83.2%) (80.5% to 85.6%)	3720 (80.2%)% (79.0% to 81.3%)	783 (81.6%) (79.1% to 84.0%)	7504 (78.8%) (78.0% to 79.6%)	8534 (77.6%) (76.8% to 78.3%)	4100 (73.2%) (72.0% to 74.3%)	3244 (79.0%) (77.8% to 80.2%)
B: Blood and blood-forming organs								
Dispensed in the year prior to pregnancy	909 (41.1%) (39.1% to 43.2%)	319 (39.0%) (35.8% to 42.4%)	1690 (36.4%) (35.0% to 37.8%)	386 (40.3%) (37.2% to 43.4%)	3379 (35.5%) (34.5% to 36.5%)	3653 (33.2%) (32.3% to 34.1%)	1518 (27.1%) (25.9% to 28.3%)	1507 (36.7%) (35.2% to 38.2%)
Dispensed during pregnancy	1426 (64.5%) (62.4% to 66.4%)	579 (70.9%) (67.7% to 73.9%)	3098 (66.8%) (65.4% to 68.1%)	658 (68.6%) (65.6% to 71.5%)	6290 (66.1%) (65.1% to 67.0%)	6637 (60.3%) (59.4% to 61.2%)	2978 (53.2%) (51.9% to 54.5%)	2582 (62.9%) (61.4% to 64.4%)
C: Cardiovascular system								
Dispensed in the year prior to pregnancy	154 (7.0%) (6.0% to 8.1%)	39 (4.8%) (3.5% to 6.5%)	269 (5.8%) (5.2% to 6.5%)	46 (4.8%) (3.6% to 6.3%)	489 (5.1%) (4.7% to 5.6%)	680 (6.2%) (5.7% to 6.6%)	374 (6.7%) (6.1% to 7.4%)	237 (5.8%) (5.1% to 6.5%)
Dispensed during pregnancy	190 (8.6%) (7.5% to 9.8%)	71 (8.7%) (6.9% to 10.3%)	410 (8.8%) (8.1% to 9.7%)	70 (7.3%) (5.8% to 9.1%)	718 (7.5%) (7.0% to 8.1%)	933 (8.5%) (8.0% to 9.0%)	438 (7.8%) (7.1% to 8.6%)	345 (8.4%) (7.6% to 9.3%)
D: Dermatologicals								
Dispensed in the year prior to pregnancy	895 (40.5%) (38.4% to 42.5%)	319 (39.0%) (35.8% to 42.4%)	1973 (42.5%) (41.1% to 43.9%)	368 (38.4%) (35.3% to 41.5%)	3404 (35.8%) (34.8% to 36.7%)	4489 (40.8%) (39.9% to 41.7%)	2421 (43.2%) (41.9% to 44.5%)	1726 (42.0%) (40.5% to 43.6%)
Dispensed during pregnancy	798 (36.1%) (34.1% to 38.1%)	314 (38.4%) (35.2% to 41.8%)	1855 (40.0%) (38.6% to 41.4%)	337 (35.1%) (32.2% to 38.2%)	3087 (32.4%) (31.5% to 33.4%)	4107 (37.3%) (36.4% to 38.2%)	2192 (39.1%) (37.9% to 40.4%)	1557 (37.9%) (36.5% to 39.4%)
G: Genitourinary system and sex hormones								
Dispensed in the year prior to pregnancy	1020 (46.1%) (44.0% to 48.2%)	394 (48.2%) (44.8% to 51.7%)	2478 (53.4%) (52.0% to 54.8%)	458 (47.8%) (44.6% to 50.9%)	5034 (52.9%) (51.9% to 53.9%)	6334 (57.6%) (56.6% to 58.5%)	3277 (58.5%) (57.2% to 59.8%)	2138 (52.1%) (50.6% to 53.6%)
Dispensed during pregnancy	788 (35.6%) (33.7% to 37.6%)	340 (41.6%) (38.3% to 45.0%)	1887 (40.7%) (39.3% to 42.1%)	351 (36.6%) (33.6% to 39.7%)	3569 (37.5%) (36.5% to 38.5%)	4606 (41.9%) (40.9% to 42.8%)	2366 (42.2%) (40.9% to 43.5%)	1579 (38.5%) (37.0% to 40.0%)
H: Systemic hormonal preparations, excluding sex hormones and insulins								
Dispensed in the year prior to pregnancy	470 (21.2%) (19.6% to 23.0%)	213 (26.1%) (23.2% to 29.2%)	1350 (29.1%) (27.8% to 30.4%)	255 (26.6%) (23.9% to 29.5%)	2473 (26.0%) (25.1% to 26.9)	3118 (28.3%) (27.5% to 29.2%)	1487 (26.5%) (25.4% to 27.7%)	1136 (27.7%) (26.3% to 29.1%)
Dispensed during pregnancy	246 (11.1%) (9.9% to 12.5%)	111 (13.6%) (11.4% to 16.1%)	687 (14.8%) (13.8% to 15.9%)	153 (16.0%) (13.8% to 18.4%)	1336 (14.0%) (13.3% to 14.7%)	1609 (14.6%) (14.0% to 15.3%)	709 (12.7%) (11.8% to 13.6%)	601 (14.6%) (13.6% to 15.8%)
J: Anti-infectives for systemic use								
Dispensed in the year prior to pregnancy	1224 (55.3%) (53.3% to 57.4%)	486 (59.5%) (56.1% to 62.8%)	2890 (62.3%) (60.9% to 63.7%)	560 (58.4%) (55.2% to 61.5%)	5284 (55.5%) (54.5% to 56.5%)	6867 (62.4%) (61.5% to 63.3%)	3544 (63.3%) (62.0% to 64.5%)	2425 (59.1%) (57.6% to 60.6%)
Dispensed during pregnancy	949 (42.9%) (40.9% to 45.0%)	382 (46.8%) (43.4% to 50.2%)	2161 (46.6%) (45.1% to 48.0%)	393 (41.0%) (37.9% to 44.1%)	4149 (43.6%) (42.6% to 44.6%)	5066 (46.0%) (45.1% to 47.0%)	2571 (45.9%) (44.6% to 47.2%)	1938 (47.2%) (45.7% to 48.7%)
L: Antineoplastic and immunomodulating agents								
Dispensed in the year prior to pregnancy	49 (2.2%) (1.7% to 2.9%)	22 (2.7%) (1.8% to 4.0%)	110 (2.4%) (2.0% to 2.8%)	31 (3.2%) (2.3% to 4.6%)	267 (2.8%) (2.5% to 3.2%)	214 (1.9%) (1.7% to 2.2%)	90 (1.6%) (1.3% to 2.0%)	101 (2.5%) (2.0% to 3.0%)
Dispensed during pregnancy	11 (0.5%) (0.3% to 0.9%)	<10	28 (0.6%) (0.4% to 0.9%)	<10	61 (0,6%) (0.5% to 0.8%)	54 (0.5%) (0.4% to 0.6%)	25 (0.4%) (0.3% to 0.7%)	20 (0.5%) (0.3% to 0.8%)
M: Musculoskeletal system								
Dispensed in the year prior to pregnancy	746 (33.7%) (31.8% to 35.7%)	354 (43.3%) (40.0% to 46.8%)	2375 (51.2%) (49.7% to 52.6%)	394 (41.1%) (38.0% to 44.2%)	3989 (41.9%) (40.9% to 42.9%)	6201 (56.4%) (55.4% to 57.3%)	3358 (59.9%) (58.7% to 61.2%)	2015 (49.1%) (47.6% to 50.6%)
Dispensed during pregnancy	231 (10.4%) (9.2% to 11.8%)	84 (10.3%) (8.4% to 12.6%)	593 (12.8%) (11.9% to 13.8%)	81 (8.4%) (6.8% to 10.4%)	898 (9.4%) (8.9% to 10.0%)	1768 (16.1%) (15.4% to 16.8%)	1012 (18.1%) (17.1% to 19.1%)	521 (12.7%) (11.7% to 13.7%)
N: Nervous system								
Dispensed in the year prior to pregnancy	1322 (59.8%) (57.7% to 61.8%)	555 (67.9%) (64.7% to 71.0%)	3451 (74.4%) (73.1% to 75.6%)	595 (62.0%) (58.9% to 65.1%)	5797 (60.9%) (59.9% to 61.8%)	8270 (75.2%) (74.3% to 76.0%)	4331 (77.3%) (76.2% to 78.4%)	2663 (64.9%) (63.4% to 66.3%)
Dispensed during pregnancy	1140 (51.5%) (49.5% to 53.6%)	503 (61.6%) (58.2% to 64.8%)	2997 (64.6%) (63.2% to 66.0%)	517 (53.9%) (50.7% to 57.0%)	5052 (53.1%) (52.1% to 54.1%)	7300 (66.3%) (65.5% to 67.2%)	3699 (66.0%) (64.8% to 67.3%)	2501 (60.9%) (59.4% to 62.4%)
P: Antiparasitic products, insecticides and repellents								
Dispensed in the year prior to pregnancy	115 (5.2%) (4.3% to 6.2%)	33 (4.0%) (2.9% to 5.6%)	248 (5.3%) (4.7% to 6.0%)	45 (4.7%) (3.5% to 6.2%)	421 (4.4%) (4.0% to 4.9%)	685 (6.2%) (5.8% to 6.7%)	393 (7.0%) (6.4% to 7.7%)	237 (5.8%) (5.1% to 6.5%)
Dispensed during pregnancy	73 (3.3%) (2.6% to 4.1%)	28 (3.4%) (2.4% to 4.9%)	170 (3.7%) (3.2% to 4.2%)	25 (2.6%) (1.8% to 3.8%)	295 (3.1%) (2.8% to 3.5%)	428 (3.9%) (3.5% to 4.3%)	269 (4.8%) (4.3% to 5.4%)	145 (3.5%) (3.0% to 4.1%)
R: Respiratory system								
Dispensed in the year prior to pregnancy	787 (35.6%) (33.6% to 37.6%)	397 (48.6%) (45.2% to 52.0%)	2518 (54.3%) (52.8% to 55.7%)	426 (44.4%) (41.3% to 47.6%)	3949 (41.5%) (40.5% to 42.5%)	5651 (51.4%) (50.4% to 52.3%)	2967 (53.0%) (51.7% to 54.3%)	1967 (47.9%) (46.4% to 49.4%)
Dispensed during pregnancy	634 (28.7%) (26.8% to 30.6%)	316 (38.7%) (35.4% to 42.1%)	1848 (39.8%) (38.4% to 41.2%)	328 (34.2%) (31.3% to 37.3%)	3097 (32.5%) (31.6% to 33.5%)	4220 (38.3%) (37.4% to 39.3%)	2110 (37.7%) (36.4% to 38.9%)	1608 (39.2%) (37.7% to 40.7%)
S: Sensory organs								
Dispensed in the year prior to pregnancy	392 (17.7%) (16.2% to 19.4%)	166 (20.3%) (17.7% to 23.2%)	774 (16.7%) (15.6% to 17.8%)	152 (15.8%) (13.7% to 18.3%)	1321 (13.9%) (13.2% to 14.6%)	1712 (15.6%) (14.9% to 16.2%)	890 (15.9%) (15.0% to 16.9%)	785 (19.1%) (17.9% to 20.4%)
Dispensed during pregnancy	223 (10.1%) (8.9% to 11.4%)	78 (9.5%) (7.7% to 11.8%)	375 (8.1%) (7.3% to 8.9%)	83 (8.7%) (7.0% to 10.6%)	710 (7.5%) (6.9% to 8.0%)	858 (7.8%) (7.3% to 8.3%)	444 (7.9%) (7.2% to 8.7%)	381 (9.3%) (8.4% to 10.2)

Expressed in n (%) and 95% CI in brackets. Differences between healthcare and assistance occupations were considered statistically significant when there was no overlap between the 95% CIs.

ATC, Anatomical, Therapeutic and Chemical.

Analyses by employment type did not reveal marked differences except among nurses and hospital service workers. Folic acid dispensing before and early in pregnancy was significantly higher in the public sector than in the private sector for nurses, whereas the opposite was observed for hospital service workers (online supplemental table 5). The dispensing of musculoskeletal, nervous system and respiratory medications was significantly higher among nurses and healthcare assistants working in the social assistance sector than among those working in the healthcare sector (online supplemental table 6).

### Medication dispensing during pregnancy

During pregnancy, 91%–96% of women were dispensed at least one medication. While medication dispensing slightly declined among healthcare assistants and hospital service workers compared with the prepregnancy period, it remained stable in other occupations. The mean number of medications dispensed was similar across occupations (mean: 8) (table 2). Occupational differences in therapeutic class exposure were less pronounced during pregnancy, but patterns persisted for nervous system, musculoskeletal and respiratory medications, which remained more frequently dispensed to social workers, psychologists, healthcare assistants and hospital service workers (table 3). Conversely, medications from alimentary tract and metabolism (ATC A) and blood and blood-forming organs (ATC B) were dispensed significantly less frequently to hospital service workers (73% (95% CI 72% to 74%) and 53% (95% CI 52% to 55%), respectively) compared with up to 82% (95% CI 79% to 84%) and 69% (95% CI 66% to 72%) among rehabilitation professionals. No significant differences were observed by employment type or sector (online supplemental tables 5 and 6).

### Specific medication classes

Folic acid dispensing from before pregnancy to the first trimester of pregnancy varied by occupation (figure 2a, online supplemental table 7), with significantly higher rates among physicians (51% (95% CI 49% to 53%)), psychologists (50% (95% CI 47% to 54%)) and rehabilitation professionals (53% (95% CI 50% to 57%)) compared with healthcare assistants (39% (95% CI 37.7% to 39.5%) and hospital service workers (30% (95% CI 29% to 32%)). Compared with non-healthcare workers with a similar socioeconomic status, most healthcare professionals received more folic acid dispensing, with the exception of hospital service workers. Psychotropic medication (including antidepressants and benzodiazepines) dispensing before pregnancy was significantly higher among hospital service workers (18% (95% CI 17% to 19%)), healthcare assistants (16% (95% CI 15% to 16%)) and social workers (14% (95% CI 13% to 15%)) than among physicians (11% (95% CI 10% to 13%)), rehabilitation professionals (10% (95% CI 8% to 12%)) and nurses (10% (95% CI 9% to 10%)) (figure 2b, online supplemental table 7). The former group had significantly higher psychotropic dispensing rates than non-healthcare workers with a similar socioeconomic status, while the latter group had significantly lower rates. Analgesic dispensing before pregnancy was significantly higher among hospital service workers (73% (95% CI 72% to 74%)) and healthcare assistants (70% (95% CI 70% to 71%)) than among other health professionals, with rates as low as 54% for physicians (95% CI 52% to 56%) and nurses (95% CI 53% to 55%), and among non-healthcare workers of similar socioeconomic status (68%) (figure 2c, online supplemental table 7). Nurses and healthcare assistants employed in the social assistance sector exhibited significantly higher analgesic dispensing rates than the nurses and healthcare assistants employed in the healthcare sector (online supplemental table 6).

**Figure 2:**
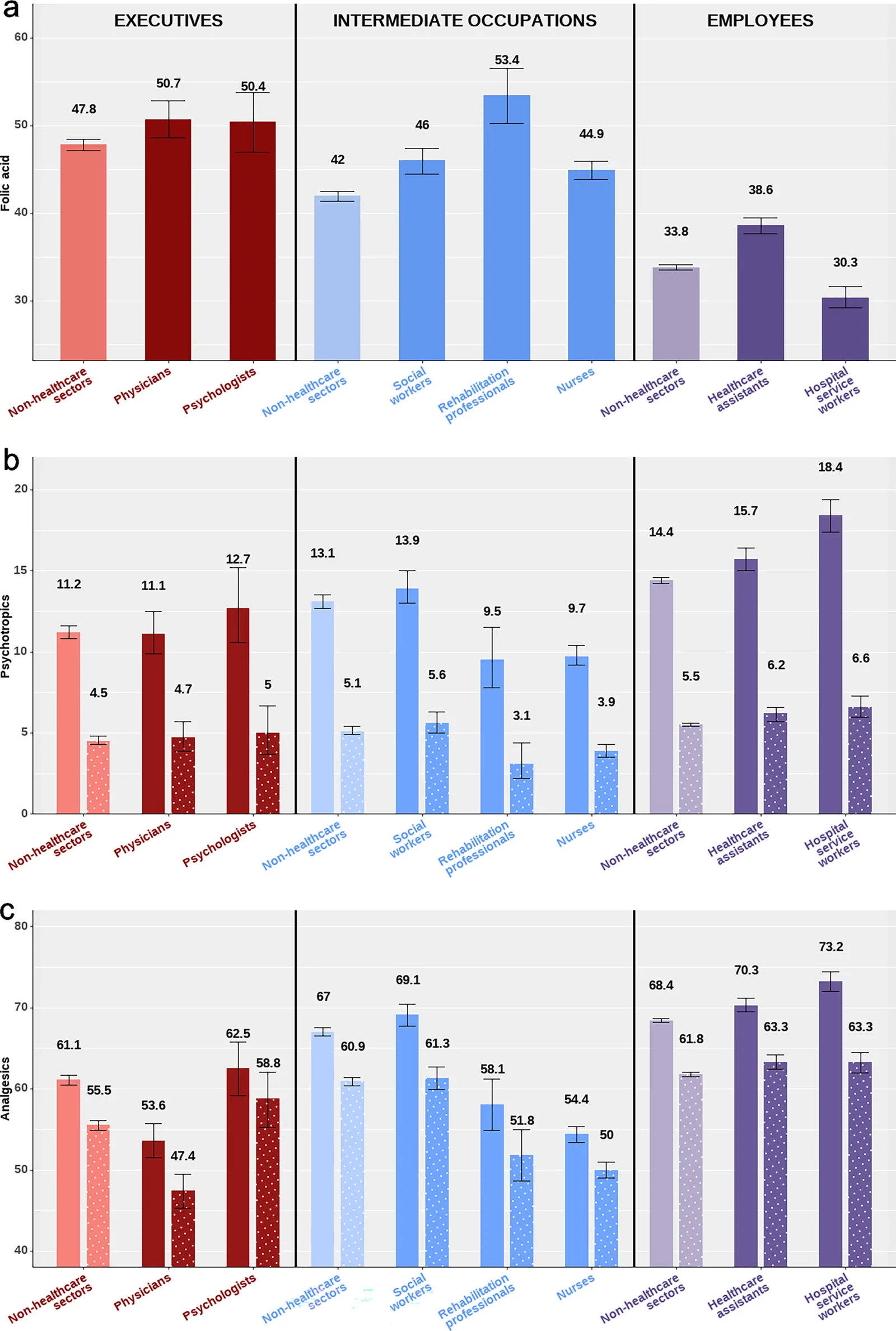
Prevalence of specific medication dispensed by occupational groups (expressed in %). (**a**) Prevalence was estimated for each occupational group from the year prior to pregnancy to the end of the first trimester of pregnancy. (**b**) and (**c**) Bars with solid texture represent estimates in the year prior to pregnancy, while bars with white-dot texture represent estimates during pregnancy.

Differences in exposure to specific medication classes were less pronounced during pregnancy (online supplemental table 7).

## Discussion

From a large population-based study of nearly 39 000 pregnant women, the use of medication of large and diverse groups of healthcare workers (working in the healthcare and social assistance sector in France) was studied. We observed substantial heterogeneity in medication dispensing across occupations, particularly for psychotropic, analgesic and musculoskeletal medications. These differences persisted when comparing healthcare occupations with non-healthcare occupations within the same occupational category, suggesting that they may be related to working conditions, rather than socioeconomic status alone. Women in occupations characterised by higher physical and psychosocial constraints and shift work, such as hospital service workers and healthcare assistants, had significantly higher rates of psychotropics (including antidepressants and benzodiazepines), analgesics and musculoskeletal medication dispensing, whereas women in occupations characterised by late and long working hours, night work and exposure to ionising radiation, such as physicians, had significantly lower medication exposure, with the exception of vitamins and supplements.

Before pregnancy, a study found that 91% of women in France had at least one medication dispensed, with an average of 10.8 prescriptions,^15^ which is in line with our results. During pregnancy, over 90% of women in our study received at least one dispensed medication, including iron and vitamins. This is consistent with previous estimates from France,^16^ but higher than those reported in other countries such as the Netherlands (79%, including iron and vitamins),^17^ Denmark (63%, including iron and vitamins)^18^ and the USA (65%, excluding iron and vitamins).^19^ However, international comparisons should be interpreted with caution, as differences in reimbursement systems may affect prevalence estimates. Compared with results among French pregnant women,^16^ women in our study had higher dispensing rates for ATC classes A (alimentary tract and metabolism), B (blood and blood-forming organs) and R (respiratory system), but lower for classes M (musculoskeletal system) and N (nervous system), with notable variation by occupations.

These disparities likely reflect a combination of factors. Occupational exposures in healthcare, such as shift work, physical demands and emotional stress may play a central role. Occupations involving higher physical workload, psychosocial demands and shift work (such as hospital service workers and healthcare assistants) showed the highest medication exposure, consistent with previous research linking such working conditions to increased use of psychotropic, musculoskeletal and digestive medications, as well as poorer self-reported health.^20–23^ Elevated rates of psychotropic and analgesic medication dispensing among hospital service workers, psychologists and social workers in our study likely reflect cumulative occupational stress. This is in line with findings from a French workforce survey where perceived difficult working conditions were associated with increased use of anxiolytic, psychotropic, musculoskeletal and digestive medications.^24^

A socioeconomic gradient was also observed, which is consistent with previous studies. Chronic conditions are more prevalent among women from lower socioeconomic status^25^ and could partly explain their greater need for medication. Higher folic acid dispensing among physicians, rehabilitation professionals and nurses suggests a greater awareness of prenatal care recommendations, while the lower rates observed among healthcare assistants and hospital service workers point to gaps in preventive advice, despite their close involvement with the healthcare system. These findings align with those of studies linking low levels of health literacy to higher rates of unplanned pregnancies, as well as lower use of folic acid before or during pregnancy.^26^ In addition, workers with lower socioeconomic status had significantly higher rates of psychotropic, analgesic and musculoskeletal medication dispensing than those from more advantaged statuses, in line with previous findings regarding both pregnant women and the working population in general.^24 27^ Our results extend these observations by demonstrating that such gradients persist even within the healthcare sector. While medication dispensing may reflect healthcare utilisation and prescribing, the contrasting patterns observed between more frequent use of symptomatic medications and lower use of preventive treatments across different occupational groups suggest that these differences may also reflect variations in health needs and preventive care.

The context of pregnancy may modify these trends, as it is associated with an increase in healthcare contacts. Overall, medication dispensing declined during pregnancy, which likely reflects increased awareness of fetal risks. Nevertheless, high exposure persisted in certain occupational groups, possibly due to a higher burden of pre-existing conditions or limited access to non-pharmacological alternatives.

### Strengths and limitations

This study has several strengths, including the use of a large, national cohort linking occupational information with healthcare data. The granularity of occupational information enabled nuanced comparisons across occupational groups, highlighting why examining overall averages may obscure important heterogeneity: aggregating all healthcare professionals would miss the higher medication exposure in vulnerable occupational subgroups. The linkage to the SNDS provided robust and detailed data on medication dispensing. However, several limitations should be acknowledged. First, medication data were limited to prescribed, reimbursed and dispensed outpatient prescriptions, and did not capture OTC medications or in-hospital medications. We cannot rule out a differential use of OTC medications among the healthcare occupations, due to differences in pharmacological knowledge and/or financial resources. Second, medication dispensing data do not necessarily reflect actual medication need and consumption. Consequently, observed differences across occupational groups may reflect not only differences in underlying morbidity, but also prescribing practices and healthcare-seeking behaviour. Third, differences were assessed using non-overlapping CIs, a conservative approach that may have missed small effects, although such differences would be unlikely to translate into clinically meaningful effects. Fourth, some healthcare occupations may influence medication dispensing patterns through specific professional roles (ie, prescriber status), while direct workplace access to medications may lead to under-recording in administrative data. Fifth, we lacked detailed information on working conditions, such as shift work or physically demanding activities, which may have contributed to health differences. Sixth, the data were collected more than 10 years ago. However, the main patterns are likely to remain valid. Nevertheless, given the worsening of working conditions in healthcare sectors in recent years, our findings are unlikely to be attenuated in more recent cohorts. Finally, because our study included mainly salaried women, our results may not be generalisable to self-employed workers.

### Perspectives for future research and implications

Future studies should explore how specific work conditions (shift work, workload, exposure to infectious agents and ergonomic constraints) contribute to the disparities in maternal health and pregnancy outcomes among healthcare professionals. Our findings support the need for targeted preventive strategies for healthcare and social assistance workers, particularly in high-risk occupations like hospital service workers and assistants. Interventions should address working conditions, improve access to preventive care and tailor occupation-specific support programmes to reduce intrasector disparities.

## Conclusion

In this large French population-based study of nearly 40 000 pregnant women working in the healthcare and social assistance sector, we observed marked occupational gradients in medication dispensing: occupations involving a heavier physical workload, high psychosocial demands and shift work (such as hospital service workers and healthcare assistants) were associated with higher exposure to psychotropic, analgesic and musculoskeletal medications and lower exposure to folic acid. These differences persisted when comparing occupations within the same socioeconomic category, suggesting that they may be related to occupational factors, such as working conditions, rather than socioeconomic status alone. Further studies will examine how work conditions impact the outcome of their pregnancy.

## Supplementary material

10.1136/oemed-2026-110920 online supplemental file 1

## Data Availability

Data may be obtained from a third party and are not publicly available.
